# Auditory-Motor Mapping Training in a More Verbal Child with Autism

**DOI:** 10.3389/fnhum.2017.00426

**Published:** 2017-09-04

**Authors:** Karen V. Chenausky, Andrea C. Norton, Gottfried Schlaug

**Affiliations:** ^1^Music, Neuroimaging, and Stroke Recovery Laboratory, Department of Neurology, Beth Israel Deaconess Medical Center Boston, MA, United States; ^2^Department of Neurology, Harvard Medical School Boston, MA, United States

**Keywords:** autism, speech therapy, intonation, AMMT, minimally verbal, speech development

## Abstract

We tested the effect of Auditory-Motor Mapping Training (AMMT), a novel, intonation-based treatment for spoken language originally developed for minimally verbal (MV) children with autism, on a more-verbal child with autism. We compared this child’s performance after 25 therapy sessions with that of: (1) a child matched on age, autism severity, and expressive language level who received 25 sessions of a non-intonation-based control treatment Speech Repetition Therapy (SRT); and (2) a matched pair of MV children (one of whom received AMMT; the other, SRT). We found a significant Time × Treatment effect in favor of AMMT for number of Syllables Correct and Consonants Correct per stimulus for both pairs of children, as well as a significant Time × Treatment effect in favor of AMMT for number of Vowels Correct per stimulus for the more-verbal pair. Magnitudes of the difference in post-treatment performance between AMMT and SRT, adjusted for Baseline differences, were: (a) larger for the more-verbal pair than for the MV pair; and (b) associated with very large effect sizes (Cohen’s *d* > 1.3) in the more-verbal pair. Results hold promise for the efficacy of AMMT for improving spoken language production in more-verbal children with autism as well as their MV peers and suggest hypotheses about brain function that are testable in both correlational and causal behavioral-imaging studies.

## Introduction

Autism spectrum disorder (ASD), a neurodevelopmental condition affecting approximately 1/68 children (Christensen et al., 2016), is characterized by: (1) impairment in social communication; and (2) the presence of repetitive behaviors or restricted interests (American Psychiatric Association and American Psychiatric Association. Task Force on DSM-5, 2013). While many children with ASD have language within the normal range (Kjelgaard and Tager-Flusberg, 2001; Tager-Flusberg et al., 2005), Rapin et al. (2009) identified two subgroups of children with ASD and impaired expressive language: one whose receptive language is also impaired, and the other whose receptive language is intact. In addition, 25%–46% of children who receive a diagnosis of ASD remain minimally verbal (MV) past age five (Tager-Flusberg et al., 2005; Kasari et al., 2013; Tager-Flusberg and Kasari, 2013; Rose et al., 2016) meaning that they have an expressive vocabulary smaller than 20 words and no word combinations (Kasari et al., 2013). Thus, spoken language treatment is important for many children with ASD.

In contrast to language skills, which are severely disordered in minimally-verbal children with ASD, musical ability may be relatively intact. For example, Applebaum et al. (1979) compared the ability of three autistic teens with no musical training to that of three typically-developing teens with considerable musical experience (e.g., at least 4 years of piano lessons) on a musical perception/production task. Although participants were matched in age, the average IQ of the autistic participants was 68, more than two standard deviations below average. Each participant was asked to imitate tone sequences of increasing complexity, ranging from single pitches to sets of four tones in an atonal configuration. The autistic participants performed as well as or better than the typically-developing, musically-trained participants on 62% of trials on average (range 45%–90%). In other work, Lai et al. (2012) found no differences in parent ratings of musical affinity in MV autistic children compared to typical controls, despite the autistic participants’ severely disordered language. Music has also been used therapeutically with MV children and adults with ASD. For example, Boso et al. (2007) reported that 52 weekly, hour-long music therapy sessions were associated with both significantly improved music skills (e.g., singing melodies of differing lengths) and decreased clinical severity scores. Taken together, the relative strengths of severely-affected individuals with ASD in the areas of musical interest, ability and learning suggest that musical activities may be a productive medium through which to develop and foster communication skills for this population.

We recently introduced and demonstrated the efficacy of a novel intonation-based treatment for MV children with ASD, Auditory-Motor Mapping Training (AMMT), which has been used successfully in that population (Wan et al., 2011) and shown to outperform a non-intonation-based control treatment, Speech Repetition Therapy (SRT; Chenausky et al., 2016, 2017). AMMT is a modification of Melodic Intonation Therapy (MIT), which has been used successfully to improve speech production in left-hemisphere stroke patients with severe nonfluent aphasia (Schlaug et al., 2009, 2010; Zipse et al., 2012). In this case report, we discuss the effects of AMMT on two children with ASD, one MV and one more verbal, and compare them to matched participants receiving the control treatment. Our goal is to understand whether AMMT can produce improvements in spoken language in a more-verbal child, commensurate with those seen in MV children with ASD. We discuss the results in the context of previous imaging findings relating spoken language performance and integrity of two white-matter tracts involved in language processing.

## Materials and Methods

### Participants

Four children with ASD, all male, aged between 4 years 1 month and 6 years 7 months, participated in this study. Diagnosis was confirmed by an Autism Diagnostic Observation Schedule (ADOS; Lord et al., 1999) score greater than 12. Inclusion criteria were the ability to: (1) correctly repeat at least two speech sounds; (2) participate in table-top activities for at least 15 min at a time; (3) follow one-step commands; and (4) imitate simple gross- and oral-motor movements like clapping hands and opening mouth. Exclusion criteria included a diagnosis of a major sensorineural or developmental condition other than ASD (e.g., concomitant deafness; known genetic disorders). MV status for the two MV participants was defined as using fewer than 20 intelligible words and having no productive syntax; this was confirmed both by parent report and child performance during initial assessments. The MV participants had scores of 1 (AMMT participant) and 3 (SRT participant) for “words used and understood” on the MacArthur-Bates Communication Development Inventory (MCDI; Fenson et al., 1993) at Baseline. Neither child produced word combinations during assessment sessions, which included obtaining language samples at Baseline. By contrast, the two more-verbal children had scores of 90 (AMMT participant) and 131 (SRT participant) on the MCDI at Baseline. Both more-verbal participants produced word combinations during a language sample at Baseline: the more-verbal AMMT participant, for example, said “I want squeeze it”; the more-verbal SRT participant said “I want more”, “I want” and “I more”. According to the vocabulary and grammar benchmarks laid out in Tager-Flusberg et al. (2009), the two MV participants met criteria for the “First Words” stage (defined as at least 2–15 words and no word combinations) and the two more-verbal participants met criteria for the “Word Combinations” stage (at least 10–50 words and mean length of utterance in morphemes 1.1–2.4).

Two tests determined the number of speech sounds children were able to repeat at baseline: (1) the first two sections of the Kaufman Speech Praxis Test (KSPT; Kaufman, 1995); or (2) a phonetic inventory test where children were asked to imitate 21 consonants and 10 vowels of English. All participants were tested with the Visual Reception, Fine Motor, Receptive Language and Expressive Language subtests of the Mullen Scales of Early Learning (MSEL; Mullen, 1995). MSEL subtest scores are generally reported as *T*-scores (*μ* = 50, *σ* = 10); however, in Table 1 we report raw scores, which are more informative for this population. All raw scores in Table 1 correspond to *T-scores* < 20, classified as “very low”. Thus, though we describe them as “more verbal”, the two non-MV participants in this study still experienced significant language delays. However, they differed from the MV participants on vocabulary size, expressive language score and developmental language stage. The MV and more-verbal AMMT participants were matched to an MV and a more-verbal control participant, respectively, based on a combination of age, ADOS score, expressive language score and phonetic inventory.

**Table 1:** Participant characteristics.

	Age (year;month)	ADOS^1^	MSEL RL^2^	MSEL EL^3^	MSEL VR^4^	MSEL FM^5^	Phonetic inventory^6^	KSPT §1^7^	KSPT §2
AMMT-V	4;1	20	13	16	26	22	25	11	63
Control 1: SRT-V	5;3	19	29	18	46	39	23	11	29
Control 2: AMMT-MV	4;2	19	10	9	13	19	11	9	10
Control 3: SRT-MV	6;7	20	12	10	22	21	4	8	3

*AMMT, Auditory-Motor Mapping Training; SRT, Speech Repetition Therapy. “V” refers to the more-verbal participants and “MV” to the minimally verbal participants. ^1^ADOS: Autism Diagnostic Observation Schedule (Lord et al., 1999) score >12 to confirm ASD diagnosis. ^2^RL: Receptive Language subscale of the Mullen Early Learning Scales (MSEL). ^3^EL: Expressive Language subscale of the MSEL. ^4^VR: Visual Reception subscale of the MSEL. ^5^FM: Fine Motor subscale of the MSEL. ^6^Phonetic inventory: the number of English vowels and consonants a child was able to imitate (max = 31 phonemes). ^7^KSPT: Kaufman Speech Praxis Test (Kaufman, 1995). Max score on Section 1 = 11; max score on Section 2 = 63*.

Children were recruited from multiple autism centers serving Greater Boston. The study was carried out in accordance with the Declaration of Helsinki and with the approval of the Institutional Review Board of Beth Israel Deaconess Medical Center. Parents of all participants provided written informed consent for their child’s participation prior to enrollment. While in the study, children continued with their regular school programs but did not participate in any other speech therapy activities or new treatments outside of school.

### Treatment

In this study, we compared the recently-introduced intonation-based treatment for MV children with ASD, AMMT, which involves repetition of intoned (sung) words or phrases (Wan et al., 2011), to a non-intonation-based control treatment, SRT. The two pitches on which AMMT stimuli are intoned follow a simplified prosodic contour (stressed syllables are intoned on the higher pitch, unstressed syllables on the lower pitch), and all syllables are produced at a rate of approximately one per second. As each syllable is produced, therapist and child tap electronic drums tuned to the same two pitches. AMMT’s multimodal nature facilitates spoken language production by activating shared motor, auditory and visual neural representations of the same vocal/manual actions (Meister et al., 2003; Ozdemir et al., 2006; Lahav et al., 2007), mimicking the co-occurrence of babbling and bimanual banging seen in typical development (Iverson and Fagan, 2004; Gernsbacher et al., 2008).

While SRT uses the same stimuli and has the same basic structure as AMMT (see “Treatment Session Structure” Section, below), in SRT stimuli are spoken, not sung; and there is no tapping on drums. Previously, we showed AMMT to produce superior improvements in spoken language in MV children with ASD over SRT (Chenausky et al., 2016, 2017). Here, we investigate whether AMMT can also lead to improvements in a more-verbal child with ASD, compared to SRT.

### Stimuli

Stimuli for the MV children consisted of two sets (Trained and Untrained) of 15 familiar bisyllabic words or phrases referring to people (“mommy”), social actions (“bye-bye”) and objects (“bubbles”) common in children’s daily lives. For the more-verbal children, stimuli also consisted of two sets (Trained and Untrained) of 16 bi- or trisyllabic stimuli, again relevant to children’s daily activities (e.g., “bathroom”, “computer”, “go outside”). Trained stimuli were presented during baseline and probe assessments and practiced during therapy sessions. Untrained stimuli were presented only during baseline and probe assessments to ascertain how well children’s speech production skills generalized to unpracticed words and phrases.

### Treatment Session Structure

Treatment sessions lasted approximately 45 min/day, 5 days/week. Breaks and rewards were provided after every 5 to 10 items, as needed. In both treatments, stimuli were presented in the context of the five-step prompt hierarchy outlined below:

1. *Listening*: Therapist introduces target phrase by showing a picture and using the phrase in a semantically meaningful context: “It’s fun to blow *bubbles*”. Therapist produces target.
2. *Unison*: “Let’s do it together: *‘bubbles’*”. Therapist produces target with child.
3. *Unison fade*: “Again, together: *‘bu…’*”. Therapist produces initial portion of target with child; child continues independently.
4. *Imitation*: (a) “My turn: *‘bubbles’*”. Therapist produces target alone. (b) “Your turn:…” Child produces target independently.
5. *Cloze*: “Last time: It’s fun to blow…” Therapist presents sematic context for stimulus; child fills in the blank independently.

### Outcome Measures and Assessments

We used four outcome measures in this study. Our primary measure was Syllables Approximated per Stimulus, a global indicator of developing speech. For a syllable to be considered *approximated* (approximately correct), its initial consonant must share two of three phonetic features (manner of articulation, place of articulation and voicing) with the target consonant; and its vowel must share two features (backness, height) with the target vowel. “Backness” and “height” refer to the anterior/posterior and dorsal/ventral position of the tongue in the mouth during production of the vowel. A more detailed explanation of this rubric appears in Chenausky et al. (2016). Three additional measures were also used: Syllables Correct per Stimulus, Consonants Correct per Stimulus and Vowels Correct per Stimulus. A consonant or vowel was considered correct if it matched the target; a syllable was considered correct if its initial consonant and its vowel both matched the target. These more stringent measures assessed speech production precision and accuracy.

Note that the measures in this study differed slightly from those in previous work. In Chenausky et al. (2016), we calculated (e.g.,) the percentage of Syllables Approximated out of a total of 60 syllables in both sets of stimuli, then compared mean scores across treatment groups. Here, we compared one child to another. Because our statistical analyses required comparisons of samples with a mean and variance, we modified the measure to Syllables Approximated per Stimulus (yielding a within-subject mean and variance) and compared means between children.

### Transcription Reliability

All baseline and probe responses were phonetically transcribed and scored by coders blind to timepoint. Five Baseline sessions were administered to each child in order to establish a stable level of performance and to allow them time to acclimatize to the room and investigator. A child’s Best Baseline was defined as the pre-treatment assessment session during which he produced the most approximately-correct syllables. For no participant was the Best Baseline session the last one, so there was no improvement over the five Baseline sessions. The Best Baseline score was then compared to performance at the P25 (post 25 treatments) assessment. A reliability study, in which 10% of Baseline and probe sessions were independently transcribed by two investigators, yielded a Cohen’s *κ* = 0.547, *p* < 0.0005 and 70.1% agreement on consonants correct, and Cohen’s *κ* = 0.270, *p* < 0.0005 and 54.7% agreement for vowels correct, as reported in Chenausky et al. (2016). These figures compare favorably to those for infant babbles (Davis and MacNeilage, 1995).

### Statistical Analyses

Two-way repeated-measures analysis of variances (ANOVAs) were performed on each outcome measure with Time (Baseline vs. P25) and Stimulus Type (Trained vs. Untrained) as within-subjects factors, and Treatment (AMMT vs. SRT) as a between-subjects factor. Because some scores were significantly different between paired children at Baseline, total Baseline score was included as a covariate, where appropriate, to correct for differences in performance. Table 2 shows Baseline scores for all participants.

**Table 2:** Baseline performance.

	Syllables approximated per stimulus	Syllables correct per stimulus	Consonants correct per stimulus	Vowels correct per stimulus
AMMT-V	1.97	1.25	2.75	1.56
Control 1: SRT-V	0.97 (*p* < 0.0005^1^)	0.5 (*p* < 0.0005)	0.81 (*p* < 0.0005)	1.16 (*p* = 0.027)
Control 2: AMMT-MV	0.77	0.17	0.63	0.60
Control 3: SRT-MV	0.27 (*p* = 0.003)	0.10 (n.s.)	0.23 (*p* = 0.02)	0.23 (*p* = 0.015)

*AMMT, Auditory-Motor Mapping Training; SRT, Speech Repetition Therapy. “V” refers to the more-verbal participants and “MV” to the minimally verbal participants. ^1^p-values vs. respective AMMT participant*.

## Results

### More-Verbal Participants

#### Syllables Approximated per Stimulus

Adjusted for Baseline performance, there was a significant main effect of Time on Syllables Approximated per Stimulus, *F*_(1,29)_ = 5.677, *p* = 0.024. Mean number of Syllables Approximated per Stimulus, averaged over both of the more-verbal participants and both stimulus types and adjusted for Baseline performance, increased over 25 sessions. There were no other significant main effects or two- or three-way interactions for this measure; however, the Time × Treatment interaction approached significance, *F*_(1,29)_ = 3.251, *p* = 0.082. The more-verbal AMMT participant improved by an adjusted mean of 0.3 Syllables Approximated per Stimulus, while the more-verbal SRT participant decreased by an adjusted mean of 0.1.

#### Syllables Correct per Stimulus

Adjusted for Baseline performance, there was a significant main effect of Time on Syllables Correct per Stimulus, *F*_(1,29)_ = 15.704, *p* < 0.0005. Mean number of Syllables Correct per Stimulus, averaged over both more-verbal participants and both stimulus types and adjusted for Baseline performance, increased over 25 sessions. There were no other significant main effects for this measure.

There was a significant Time × Treatment interaction for Syllables Correct per Stimulus, adjusted for Baseline performance, *F*_(1,29)_ = 27.787, *p* < 0.0005, Cohen’s *d* = 1.92 (very large). The more-verbal AMMT participant improved by an adjusted mean of 0.7 Syllables Correct per Stimulus, while the more-verbal SRT participant decreased by an adjusted mean of 0.1. There were no other significant 2-way interactions; however, there was a significant Time × Stimulus × Treatment interaction, *F*_(1,29)_ = 5.052, *p* = 0.032. The more-verbal AMMT participant improved by an adjusted mean of 0.5 Syllables Correct per Trained Stimulus and by 0.9 Syllables Correct per Untrained Stimulus. The more-verbal SRT participant improved by an adjusted mean of 0.1 Syllables Correct per Trained Stimulus and decreased by 0.3 Syllables Correct per Untrained Stimulus.

#### Consonants Correct per Stimulus

Adjusted for Baseline performance, there was a significant main effect of Time on Consonants Correct per Stimulus, *F*_(1,29)_ = 13.505, *p* = 0.001). Mean number of Consonants Correct per Stimulus, averaged over both more-verbal participants and both stimulus types and adjusted for Baseline performance, increased over 25 sessions. There were no other significant main effects for this measure.

There was a significant Time × Treatment interaction for Consonants Correct per Stimulus, adjusted for Baseline performance, *F*_(1,29)_ = 18.203, *p* < 0.0005, Cohen’s *d* = 1.56 (very large). The more-verbal AMMT participant improved by an adjusted mean of 1.2 Consonants Correct per Stimulus, while the more-verbal SRT participant decreased by an adjusted mean of 0.4. There were no other significant two- or three-way effects for this measure.

#### Vowels Correct per Stimulus

Adjusted for Baseline performance, there was a significant main effect of Time on Vowels Correct per Stimulus, *F*_(1,29)_ = 21.657, *p* < 0.0005. Mean number of Vowels Correct per Stimulus, averaged over both more-verbal participants and both stimulus types and adjusted for Baseline performance, increased over 25 sessions. There were no other significant main effects for this measure.

There was a significant Time × Treatment effect on Vowels Correct per Stimulus, adjusted for Baseline performance, *F*_(1,29)_ = 13.663, *p* = 0.001, Cohen’s *d* = 1.35 (very large). The more-verbal AMMT participant improved by an adjusted mean of 0.5 Vowels Correct per Stimulus, while the more-verbal SRT participant decreased by an adjusted mean of 0.3, over 25 sessions. There were no other significant two- or three-way interactions for this measure.

**Figure 1:**
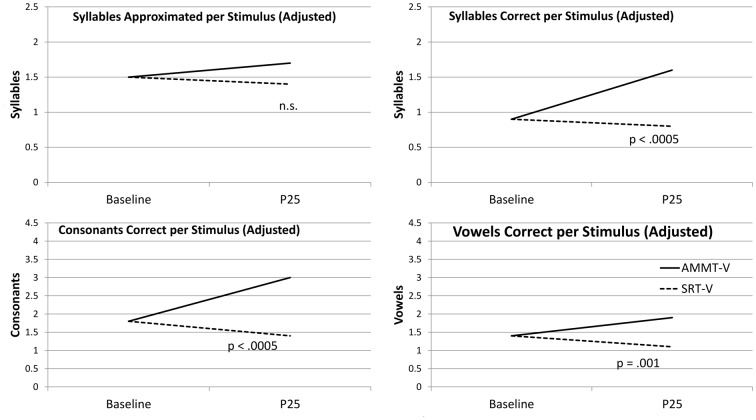
Change over Time (Verbal Participants). AMMT-V, More-verbal Auditory-Motor Mapping Training participant; SRT-V, More-verbal Speech Repetition Therapy participant.

Figure 1 shows the change over time on each measure for the two more-verbal participants, adjusted for Baseline performance.

### Minimally Verbal (MV) Participants

#### Syllables Approximated per Stimulus

Adjusted for Baseline performance, there was a significant main effect of Time for Syllables Approximated per Stimulus, *F*_(1,27)_ = 4.069, *p* = 0.041, indicating that the mean number of Syllables Approximated per Stimulus, averaged over both MV participants and both stimulus types and adjusted for Baseline performance, increased over 25 sessions. There were no other significant main effects for Syllables Approximated per Stimulus.

There was a significant Time × Treatment interaction for Syllables Approximated per Stimulus, adjusted for Baseline performance, *F*_(1,27)_ = 5.362, *p* = 0.028, Cohen’s *d* = 0.88 (large). The MV AMMT participant improved by an adjusted mean of 0.6 Syllables Approximated per Stimulus from Best Baseline to P25, and the MV SRT participant improved by an adjusted mean of only 0.1 over the same time period. There were no other significant two- or three-way effects for Syllables Approximated per Stimulus.

#### Syllables Correct per Stimulus

Unadjusted for Baseline performance, there was a significant main effect of Time on Syllables Correct per Stimulus, *F*_(1,28)_ = 16.141, *p* < 0.0005. Mean number of Syllables Correct per Stimulus, averaged over both MV participants and both stimulus types, increased over 25 sessions. There were no other significant main effects for this measure.

There was also a significant Time × Treatment interaction for Syllables Correct per Stimulus, unadjusted for Baseline performance, *F*_(1,28)_ = 5.271, *p* = 0.029, Cohen’s *d* = 0.87 (large). The MV AMMT participant improved by a mean of 0.3 Syllables Correct per Stimulus, and the MV SRT participant by a mean of 0.1 Syllables Correct per Stimulus, over 25 sessions.

#### Consonants Correct per Stimulus

Adjusted for Baseline performance, there was a significant main effect of Time for Consonants Correct per Stimulus, *F*_(1,27)_ = 8.056, *p* = 0.009. Mean number of Consonants Correct per Stimulus, averaged over both MV participants and both stimulus types and adjusted for Baseline performance, increased over 25 sessions. There were no other significant main effects for this measure.

There was also a significant Time × Treatment interaction for Consonants Correct per Stimulus, adjusted for Baseline performance, *F*_(1,27)_ = 5.726, *p* = 0.024, Cohen’s *d* = 0.9 (large). The MV AMMT participant improved by an adjusted mean of 0.7 Consonants Correct per Stimulus over 25 sessions, compared to an adjusted mean of 0.1 for the MV SRT participant. There were no other significant two- or three-way interactions for this measure.

#### Vowels Correct per Stimulus

Adjusted for Baseline performance, there was a significant main effect of Time for Vowels Correct per Stimulus, *F*_(1,27)_ = 4.506, *p* = 0.043. In this case, both MV children improved on this measure over 25 sessions, the MV AMMT participant by 0.5 Vowels per Stimulus and the MV SRT participant by 0.4 (between-treatment difference n.s.). There were no other significant main effects, and no significant two- or three-way interactions, for this measure.

**Figure 2:**
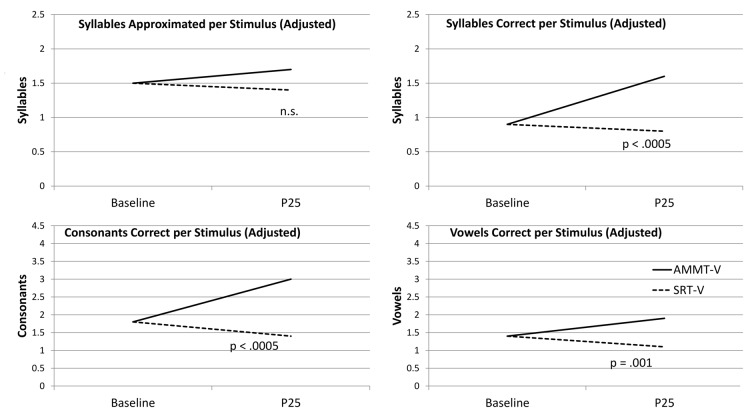
Change Over Time (Minimally verbal (MV) Participants). AMMT-MV, Minimally verbal Auditory-Motor Mapping Training participant; SRT-MV, Minimally verbal Speech Repetition Therapy participant.

Figure 2 shows the change over time on each measure for the two MV participants, adjusted for Baseline performance where appropriate.

## Discussion

In this study, we assessed the effect of AMMT, an intonation-based spoken language treatment with proven efficacy as an intervention for MV children with ASD (Wan et al., 2011; Chenausky et al., 2016), on a more-verbal child with ASD. We compared his progress to a matched participant who received SRT and to a matched pair of MV children (one who received AMMT; the other, SRT) with ASD. Several results emerged.

First, AMMT resulted in a greater improvement in most of our outcome measures than SRT for our more-verbal participant, when differences in Baseline performance were taken into account. The more-verbal AMMT participant also experienced a comparatively greater improvement than the more-verbal SRT participant in number of Syllables, Consonants and Vowels Correct per Stimulus, over 25 treatment sessions.

Second, the adjusted improvements seen in AMMT over SRT for Syllables Correct, Consonants Correct and Vowels Correct per Stimulus for the more-verbal participant with ASD are associated with very large effect sizes (Cohen’s *d* > 1.3). These effect sizes were larger than those for the difference between the MV AMMT and SRT participants (Cohen’s *d* approximately 0.9). Together, these results suggest that AMMT may be at least as effective for more-verbal children with ASD as it is for MV children with ASD.

The finding of no significant between-treatment difference in Syllables Approximated per Stimulus suggests that this measure is too coarse-grained for the speech of children with better-than-minimal spoken language. The more stringent outcome measure of Syllables Correct may be more useful for this group of children. Finally, though the differential findings on Trained and Untrained Stimuli for the more-verbal AMMT and SRT participants may be child-specific, it is also possible they may have arisen from differences in phonetic complexity or familiarity of the words/phrases between the two stimulus sets. Alternatively, they may suggest that AMMT promotes greater skill generalization than SRT. This would be an important finding for children with ASD, who struggle with generalizing skills to new contexts (Plaisted, 2001; Happé and Frith, 2006).

In addition to describing the behavioral results of 25 treatment sessions of AMMT, it is also worth considering the neural substrates likely to be involved in the improvements seen. AMMT is hypothesized to work by engaging a network of brain regions that are activated by auditory, motor and visual actions (Wan et al., 2011). Lai et al. (2012) showed that, while listening to speech, activation in the left inferior frontal gyrus (IFG) was lower in children with ASD compared to typically developing controls, yet it was higher than in controls while listening to song, even in the presence of reduced integrity of the left arcuate fasciculus (AF) in the children with ASD. This suggests that music-making activities and intonation may be a unique vehicle for engaging the IFG in two of its presumed functions—the mapping of sounds to articulatory actions and their sequential execution. The AF connects auditory-perceptual regions in the temporal lobe to motor-related regions in the posterior inferior portion of the frontal lobe (Catani et al., 2005). As such, it is thought to be responsible for the bidirectional mapping of speech articulation and acoustics (Saur et al., 2008; Leclercq et al., 2010), to mediate new word learning (López-Barroso et al., 2013), and to form part of the dorsal language pathway (Friederici, 2011). Further research suggests that integrity of the AF and the extreme capsule fiber tract (EmC), another white-matter tract involved in language comprehension and production, may be related to AMMT participants’ improvements in spoken language production. The extreme capsule fiber tract (EmC) links the more anterior portion of the IFG to the middle-posterior portion of the superior temporal gyrus (Makris and Pandya, 2009), assuming a slightly more ventral course for this tract near the insular cortex. Although the functions of the EmC are less clear, it has been proposed that this tract is involved in the comprehension (Saur et al., 2008) and production of morphologically more complex words (Rolheiser et al., 2011).

In previous work (Chenausky et al., 2017) we showed that integrity of the left AF, measured at baseline, predicted improvement in percent syllable-initial consonants correct after 25 sessions of AMMT in a group of 10 MV children with ASD. In ongoing work in our lab, we have found that when FA of the language tracts was relativized by that of the corticospinal tract (CST)—a motor execution network that has been shown to be compromised in ASD (Carper et al., 2015)—relative integrity of the right EmC and both the right and left AF at baseline predicted improvement in percent syllables approximately correct, and relative integrity of the right EmC at baseline predicted improvement in percent vowels correct.

Taken together, the behavioral results reported here, combined with previously identified links between imaging and behavioral findings, lead to empirically testable hypotheses regarding the neural substrates required for more-verbal children with ASD to benefit from an intonation-based treatment. Specifically, we would predict that, in this population as in the MV group, the degree of improvement in consonant production should be positively related to integrity of the left AF, improvement in vowel production to integrity of the right EmC, and the degree of improvement in syllables correct should be positively related to the integrity of both tracts. Furthermore, the behavioral findings reported on here may support the hypothesis, put forth in Hardy and LaGasse (2013), that external rhythmic, musical cues may provide useful templates for organizing motor output in children with ASD by: (1) decreasing motor planning demands; and (2) increasing movement efficiency and accuracy. Additional support for this hypothesis, as well as for the idea that music is beneficial for children with ASD for reasons beyond its ability to increase motivation and attention, comes from research showing that MIT, the treatment from which AMMT was derived, has been shown to be effective for improving verbal output in patients with moderate to severe nonfluent aphasia after a left-hemisphere stroke (Schlaug et al., 2009, 2010; Zipse et al., 2012; Wan et al., 2014).

## Concluding Remarks

AMMT holds promise for improving the spoken language of children with ASD who are not considered MV, but who still struggle with significant expressive language and speech-production deficits. The current results should be replicated in a larger group of more-verbal children with ASD. In addition, pre- and post-treatment imaging studies should be performed to both test whether integrity of the left AF and the right EmC are positive predictors of the degree to which more-verbal children with ASD can improve in spoken language production, and to verify whether integrity of those two tracts does, in fact, increase as children’s spoken language abilities improve.

## Author Contributions

GS, KVC and ACN: substantial contributions to the conception or design of the work; or the acquisition, analysis, or interpretation of data for the work. KVC, GS and ACN: drafting the work or revising it critically for important intellectual content. KVC, GS and ACN: final approval of the version to be published. GS, KVC and ACN: agreement to be accountable for all aspects of the work in ensuring that questions related to the accuracy or integrity of any part of the work are appropriately investigated and resolved.

## Conflict of Interest Statement

The authors declare that the research was conducted in the absence of any commercial or financial relationships that could be construed as a potential conflict of interest. The handling Editor currently co-hosts a Research Topic with one of the authors GS, and confirms the absence of any other collaboration. He states that the process met the standards of a fair and objective review.
